# Replacement of the Endogenous Starch Debranching Enzymes ISA1 and ISA2 of Arabidopsis with the Rice Orthologs Reveals a Degree of Functional Conservation during Starch Synthesis

**DOI:** 10.1371/journal.pone.0092174

**Published:** 2014-03-18

**Authors:** Sebastian Streb, Samuel C. Zeeman

**Affiliations:** Institute for Agricultural Sciences, Department of Biology, ETH Zurich, Zurich, Switzerland; University of Potsdam, Germany

## Abstract

This study tested the interchangeability of enzymes in starch metabolism between dicotyledonous and monocotyledonous plant species. Amylopectin - a branched glucose polymer - is the major component of starch and is responsible for its semi-crystalline property. Plants synthesize starch with distinct amylopectin structures, varying between species and tissues. The structure determines starch properties, an important characteristic for cooking and nutrition, and for the industrial uses of starch. Amylopectin synthesis involves at least three enzyme classes: starch synthases, branching enzymes and debranching enzymes. For all three classes, several enzyme isoforms have been identified. However, it is not clear which enzyme(s) are responsible for the large diversity of amylopectin structures. Here, we tested whether the specificities of the debranching enzymes (ISA1 and ISA2) are major determinants of species-dependent differences in amylopectin structure by replacing the dicotyledonous Arabidopsis isoamylases (*At*ISA1 and *At*ISA2) with the monocotyledonous rice (*Oryza sativa*) isoforms. We demonstrate that the ISA1 and ISA2 are sufficiently well conserved between these species to form heteromultimeric chimeric Arabidopsis/rice isoamylase enzymes. Furthermore, we were able to reconstitute the endosperm-specific rice *Os*ISA1 homomultimeric complex in Arabidopsis *isa1isa2* mutants. This homomultimer was able to facilitate normal rates of starch synthesis. The resulting amylopectin structure had small but significant differences in comparison to wild-type Arabidopsis amylopectin. This suggests that ISA1 and ISA2 have a conserved function between plant species with a major role in facilitating the crystallization of pre-amylopectin synthesized by starch synthases and branching enzymes, but also influencing the final structure of amylopectin.

## Introduction

Starch is the major storage carbohydrate in plants and an important renewable resource for both the food and non-food industry sectors. Starch is comprised of two glucose polymers (amylopectin and amylose) and accumulates in plant tissues as semi-crystalline granules. Amylopectin accounts for the majority of the granule mass (around 60% to 90%, depending on the botanical source). It is a branched molecule in which α-1,4-linked glucan chains are connected via α-1,6-bonds [1], [2] resulting in a tree-like structure. On average, there is one branch point every 20–25 glucose residues. However, the arrangement of branch points is thought to be non-random, such that linear chain segments can align together to form double helices that pack into stable, semi-crystalline lamellae. The branch points are concentrated in the amorphous regions between these crystalline lamellae [1].

The higher-order structures adopted by amylopectin are thought to occur in all wild-type starches and underlie the water-insoluble granular characteristics of starch. Nevertheless, there is considerable variation in starch granule morphology, structure and composition between plant sources. These factors are important due to the impact they have on starch properties, which are relevant for downstream functional applications. A detailed understanding of how different biosynthetic enzymes influence amylopectin structure has been hindered by the fact that it is currently not possible to determine the exact structure of amylopectin. Also, we can only partially relate the physical properties of starch to its structure, and hence it is difficult to predictably control these characteristics through manipulation of enzyme abundance and/or specificities.

Starch synthesis is mediated by three enzyme classes. Starch synthases extend α-1,4-linked chains by transferring new glucose units from ADP-glucose to the non-reducing end. Branch points are introduced by branching enzymes, which cleave an existing α-1,4-bond of a linear chain and transfer the cut end to another chain, creating an α-1,6-bond. Both starch synthases and branching enzymes exist as multiple isoforms, thought to have different specificities [3]–[5]. A third class, the debranching enzymes, are involved in the removal of α-1,6-bonds. This hydrolytic step is obviously required during starch re-mobilization, but is interestingly also required for normal starch synthesis [5]–[8]. Debranching enzymes can be divided into two classes: isoamylase (ISA) and limit-dextrinase (LDA). The isoamylase class is subdivided into proteins designated as ISA1, ISA2 and ISA3 [9], [10]. ISA3 and LDA are mainly involved in starch breakdown [11]–[13], whereas ISA1 and ISA2 participate in starch synthesis. Current evidences suggest that ISA1 is an active enzyme, whereas ISA2 proteins are non-catalytic due to changes in 6 out of 8 key amino acids within the active site [9], [14], [15]. In all plant species studied, ISA1 and ISA2 are found in heteromultimeric complexes, in which the ISA2 subunit is proposed to have a regulatory function or confer substrate specificity (Arabidopsis: [16]; Potato: [17]; *Chlamydomonas reinhardtii*: [18]; maize: [19]; rice: [20]). In addition, ISA1 homomultimers have been shown to occur in rice and maize endoperm [19], [20] and proposed for *C. reinhardtii* [18]. Thus far, homomultimer forms of ISA1 have not been reported in leaves. Therefore, it is still unknown whether this reflects a tissue-specific feature (heterotrophic versus autotrophic) or an evolutionary difference between monocot and dicot plants.

The role of these isoamylase complexes in starch biosynthesis has been determined through phenotypic analysis in a range of plant species and tissues in which *ISA1* or *ISA2* gene expression has been reduced or abolished. The loss of ISA1 results in a reduction of granular starch in endosperms of maize [21], rice [6] and barley [22], in *C. reinhardtii* cells [18], [23], [24], in Arabidopsis leaves [13], [16] and in potato tubers [17]. In all instances, the starch was partially replaced by a water soluble glucose polymer called phytoglycogen, which had shorter chain lengths compared to amylopectin in chain length distribution (CLD) analyses, and a higher degree of branching. The impact of mutations in *ISA2* is more variable. In endosperms of maize and rice, loss of ISA2 had no measurable effect, explained by the fact that these tissues contain still the active ISA1 homomultimeric complexes [19], [25]. In Arabidopsis, loss of ISA2 causes the same phenotype as the loss of ISA1 [13], [16]. This observation is explained by the fact that Arabidopsis leaves appears to contain only the heteromultimeric ISA1/ISA2 complex, and the loss of either protein subunit results in a loss of the enzymatic activity destabilization of the remaining subunit [15]. Overall, the differing phenotypic severity caused by the loss of ISA1 and the existence of both homomultimeric ISA1 and heteromultimeric ISA1/ISA2 complexes means that function of isoamylases in amylopectin biosynthesis is still not fully understood.

In this study, we wanted to address three hypotheses arising from our current knowledge. First, are differences in starch structure between different plant species due to different catalytic specificities of ISA1 and ISA2 protein complexes? Second, are the functions between the ISA complexes from different plant species conserved? Third, are the subunits of the ISA complexes adequately conserved such that they are interchangeable between species? To answer these questions we replaced the endogenous ISA proteins in the dicot Arabidopsis (*At*ISA1 and *At*ISA2) with the respective monocot rice isoforms (*Os*ISA1 and *Os*ISA2). Our data show that active heteromultimeric complexes can be formed between *At*ISA1 and *Os*ISA2 as well as *At*ISA2 and *Os*ISA1. Additionally, we were able to reconstitute the *Os*ISA1 homomultimeric complex as found in rice endosperm. The resulting transgenic lines synthesize comparable amounts of starch to wild-type Arabidopsis plants. This illustrates that rice isoforms can replace the endogenous Arabidopsis proteins. Furthermore, in addition to playing a major role in facilitating starch biosynthesis, differences in the final structure of amylopectin between the transgenic lines suggests that the isoamylases enzymes composed of ISA1 and ISA2 subunits also contribute to the species-specific differences in amylopectin structure.

## Results

### Transient and storage starch structure in monocot and dicot plants

**Figure 1:**
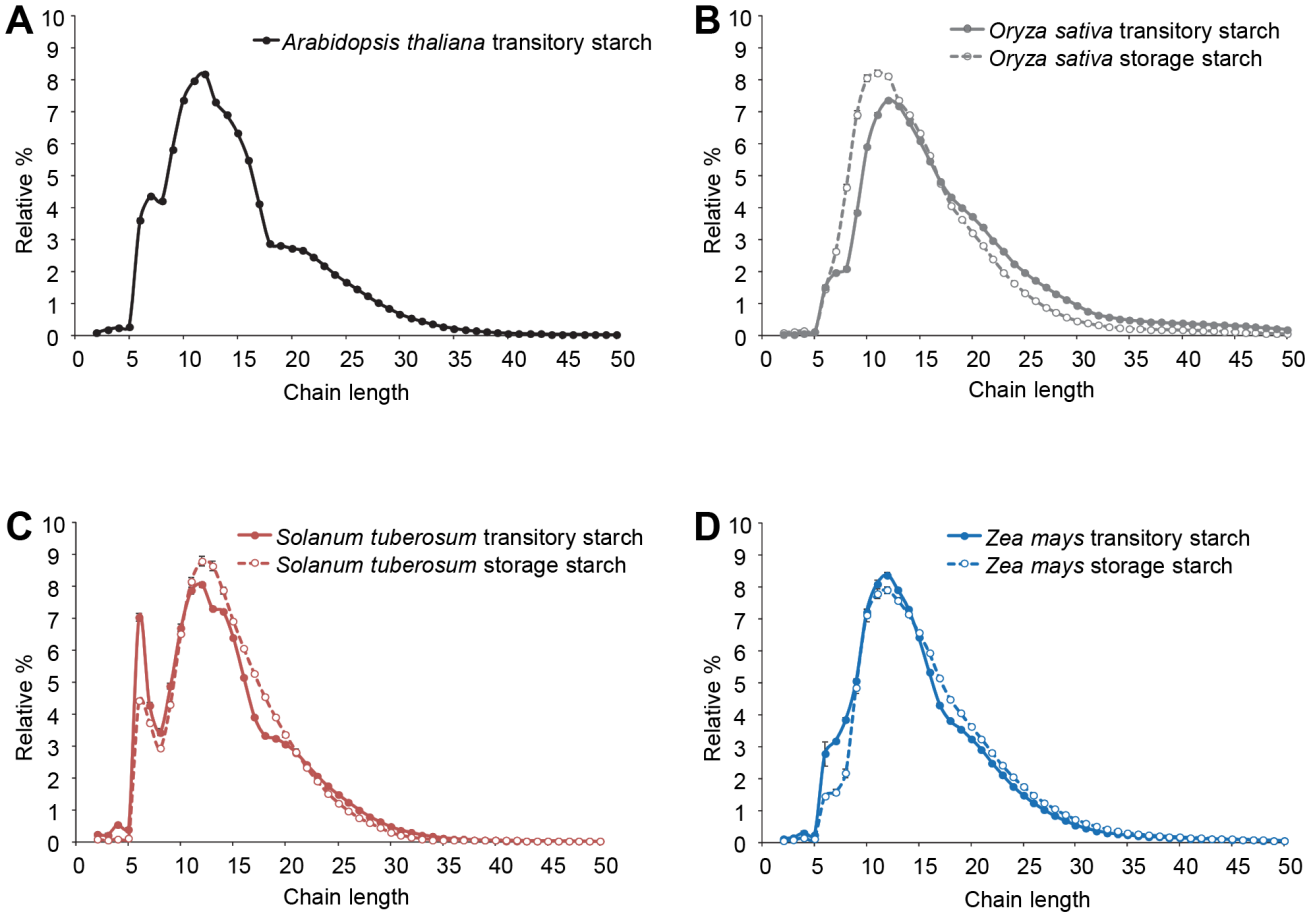
Amylopectin structure in different plant species and tissues. Comparison of chain length distributions (CLDs) of starch in different tissues from *Arabidopsis thaliana*, *Oryza sativa* (rice), *Solanum tuberosum* (potato) and *Zea mays* (maize). A) CLD of Arabidopsis leaf starch, harvested at the end of the light period. B) CLDs of rice leaf starch (filled grey circle) and seed storage starch (open grey circle). C) CLDs of potato leaf starch (filled red circle) and tuber storage starch (open red circle). D) CLDs of maize leaf starch (filled blue circle) and seed storage starch (open blue circle). Note that in all cases, the CLDs differ between the tissues from the same plant species. Values are means ± SE (n = 3).

Despite the conservation in starch biosynthetic enzymes between species (SSs, BEs and DBEs), the reported amylopectin structures differ considerably between different plant species and tissues [26]. This may be due to differences in enzyme specificities and/or relative expression levels. To identify species with significant differences in amylopectin structure and exclude variability resulting from the different techniques used between different laboratories, we analysed the structure of amylopectin from starch extracted from two monocot species (maize and rice) and two dicot species (Arabidopsis and potato). Although the plants were grown under different conditions appropriate for each species (see Material and Methods), the starch analysis was done in parallel. We used the well-established method of chain length distribution (CLD) analysis, where the α-1,6-bonds of amylopectin are enzymatically hydrolysed, resulting in linear chains that can be separated and quantified by HPAEC-PAD (high performance anion exchange chromatography with pulsed amperometric detection). The CLD profiles had distinct species-specific features and varied between tissues (Fig. 1).

In the CLDs of leaf starch from all four species, chains with a degree of polymerisation (d.p.) of 12 were most abundant, but the profiles differed in other ways. The CLD of potato, had a prominent peak of chains of d.p. 6 and d.p. 7 (Fig. 1C), which was also visible in Arabidopsis, but less prominent in maize and rice (Fig. 1). The Arabidopsis leaf starch CLD also had a discontinuity at d.p. 18, suggestive of a subpopulation of chains of between d.p. 5 and d.p. ∼20, overlapping with a population of longer chains up to d.p. 35. This CLD discontinuity was also visible for potato leaf starch but much less apparent for the monocot leaf starches. Similarly, the amylopectin structure of storage starch from the endosperms of rice and maize, and from potato tuber differed from one another (Fig. 1B-D). Potato amylopectin still had an early peak of chains at d.p. 6 and d.p. 7. This was also visible in maize, but not in rice. Otherwise, the amylopectin CLDs from the storage starches had smooth distributions up to d.p. ∼35, with no discontinuity at d.p. 18. None of the three crop species had the same amylopectin structure within their transient and storage starches (Fig. 1B-D). These structural differences between species and tissues indicate that the biosynthetic machinery in each case is not identical.

### Generation of transgenic Arabidopsis lines expressing *OsISA1* and *OsISA2*

We investigated the role of the isoamylases (ISA1 and ISA2) in determining the starch structure between plants using a gene-swap experiment. We chose the rice, which had the largest difference in starch structure compared to Arabidopsis (Fig. 1). The latter is highly amenable to genetic transformation and mutants in *ISA1* and *ISA2* have been characterised, together with the double mutant [13], [16].

We cloned the protein-coding region of *OsISA1* and *OsISA2* into the pB7WG2 plant Gateway-compatible expression vector [27]. *OsISA2* was expressed in the Arabidopsis *isa2* single mutant under the cauliflower mosaic virus (CaMV) 35S promoter. The *OsISA1* construct was transformed into the Arabidopsis *isa1* single mutant and the *isa1isa2* double mutants. In contrast to *Os*ISA2, which can only form a heteromultimeric complex with *Os*ISA1, the *Os*ISA1 protein is reported to also form a homomultimeric complex [20]. Therefore, we hypothesized that the expression of *OsISA1* might be able to complement both of the missing Arabidopsis proteins.

**Figure 2:**
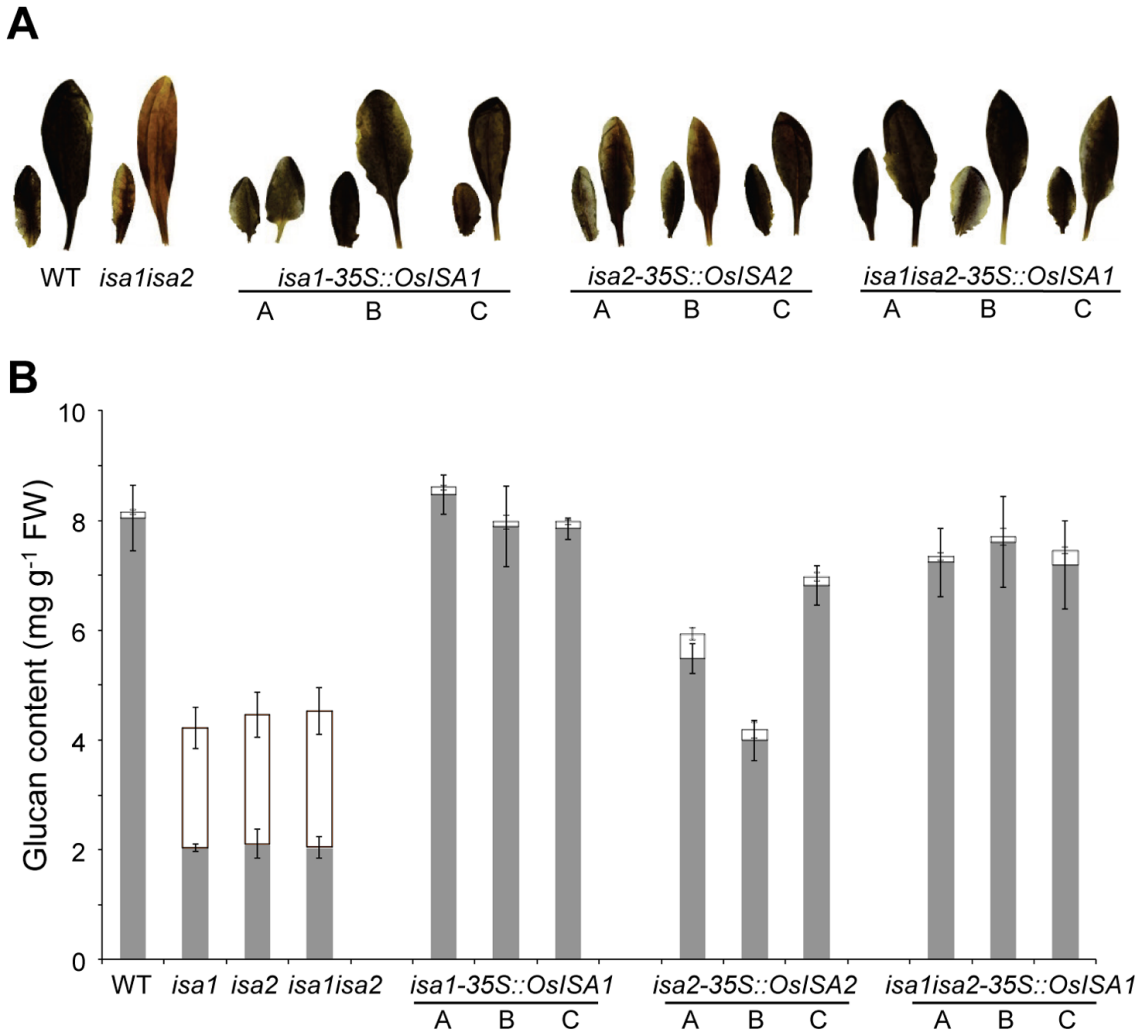
Starch content in Arabidopsis isoamylase mutants transformed with rice isoamylases. A) Arabidopsis T_1_ transformed lines expressing rice ISA1 (*OsISA1*) or rice ISA2 (*OsISA2*) in the background of *isa1*, and *isa2* single and *isa1isa2* double mutants. Qualitative iodine staining was used to isolate plants which revert the red-brown staining of the *isa1*, *isa2*, *isa1isa2* mutants back to wild-type (WT) staining. Three independent lines are shown (A, B and C), which were used in all subsequent experiments. B) Plants grown for 28 days in a 12-h light/12-h dark regime were harvested at the end of the day and analysed for insoluble starch (grey bar) and phytoglycogen (white bar). Arabidopsis *isa1* mutants complemented with *Os*ISA1 (*isa1-35S::OsISA1*), *isa2* mutants complemented with *Os*ISA2 (*isa2-35S::OsISA2*) and *isa1isa2* double mutants complemented with *Os*ISA1 alone (*isa1isa2-35S::OsISA1*). Samples comprised all the leaves from a single plant and were extracted using perchloric acid. Starch in the insoluble fraction and phytoglycogen in the soluble fraction were measured after enzymatic hydrolysis to glucose. Each point is the mean ± SE (n = 4).

The three Arabidopsis mutants used for transformation all have the same reduced starch, phytoglycogen-accumulating phenotype, due to the complete loss of the heteromultimeric isoamylase activity. The altered glucan composition of these mutants makes it possible to distinguish between them and wild-type plants by simple iodine staining. Amylopectin of wild-type plants stain dark brown whereas the phytoglycogen in the mutants stains a more red-brownish colour (Fig. 2A). We isolated a minimum of ten independent lines per transformation from the T_1_ generation. We then screened these lines by iodine staining to search for those in which the loss of the endogenous isoamylase activity was complemented by the expression of the rice gene. Half or more of the T_1_ transformants stained like the wild type, suggesting complementation. We confirmed that this phenotype was stable in the T_2_ generation of the three most promising candidate lines per genotype (Fig. 2A). These lines were analysed further and were named as follows: *isa1* mutants transformed with *OsISA1*, *isa1-35S::OsISA1* A, B and C; *isa2* mutants transformed with *OsISA2*, *isa2-35S::OsISA2* A, B and C; *isa1isa2* double mutants transformed with *OsISA1*, *isa1isa2-35S:: OsISA1* A, B and C.

### Quantitative starch and phytoglycogen measurement

Iodine staining is suitable for rapid screening but is largely qualitative. Therefore we measured the starch and phytoglycogen in all of the transformed lines at the end of a 12-h photoperiod. As expected, wild-type plants had around 8 mg starch per gram plant fresh weight and contained only trace amounts of soluble glucan. In contrast, the *isa1*, *isa2* and *isa1isa2* mutants contained both starch and phytoglycogen and synthesized less glucan in total than the wild type (Fig. 2B). All three mutants had the same phenotype, as reported earlier [13], [16]. The starch measurements for the three *isa1-35S::OsISA1* lines confirmed the results from the iodine staining (Fig. 2A) as they contained wild-type levels of starch and no phytoglycogen. Interestingly, full complementation was also achieved in the *isa1isa2-35S::OsISA1* plants despite the fact that no ISA2 protein is present (neither *At*ISA2 nor *Os*ISA2). Comparable results were also recently described for these *isa* mutants transformed with the Maize *ISA1* gene [28].

The three *isa2-35S::OsISA2* lines showed a marked change in phenotype compared with the parental line, but complementation was incomplete. Although all three lines contained only starch, with little or no phytoglycogen, the starch content varied significantly between the lines (Fig. 2B). The line *isa2-35S::OsISA2* B contained only half as much starch as the wild-type (comparable to the sum of starch and phytoglycogen in the *isa2* parental line), whereas *isa2-35S::OsISA2* C contained almost wild-type amounts of starch.

### Rice isoamylase complex formation in Arabidopsis

**Figure 3:**
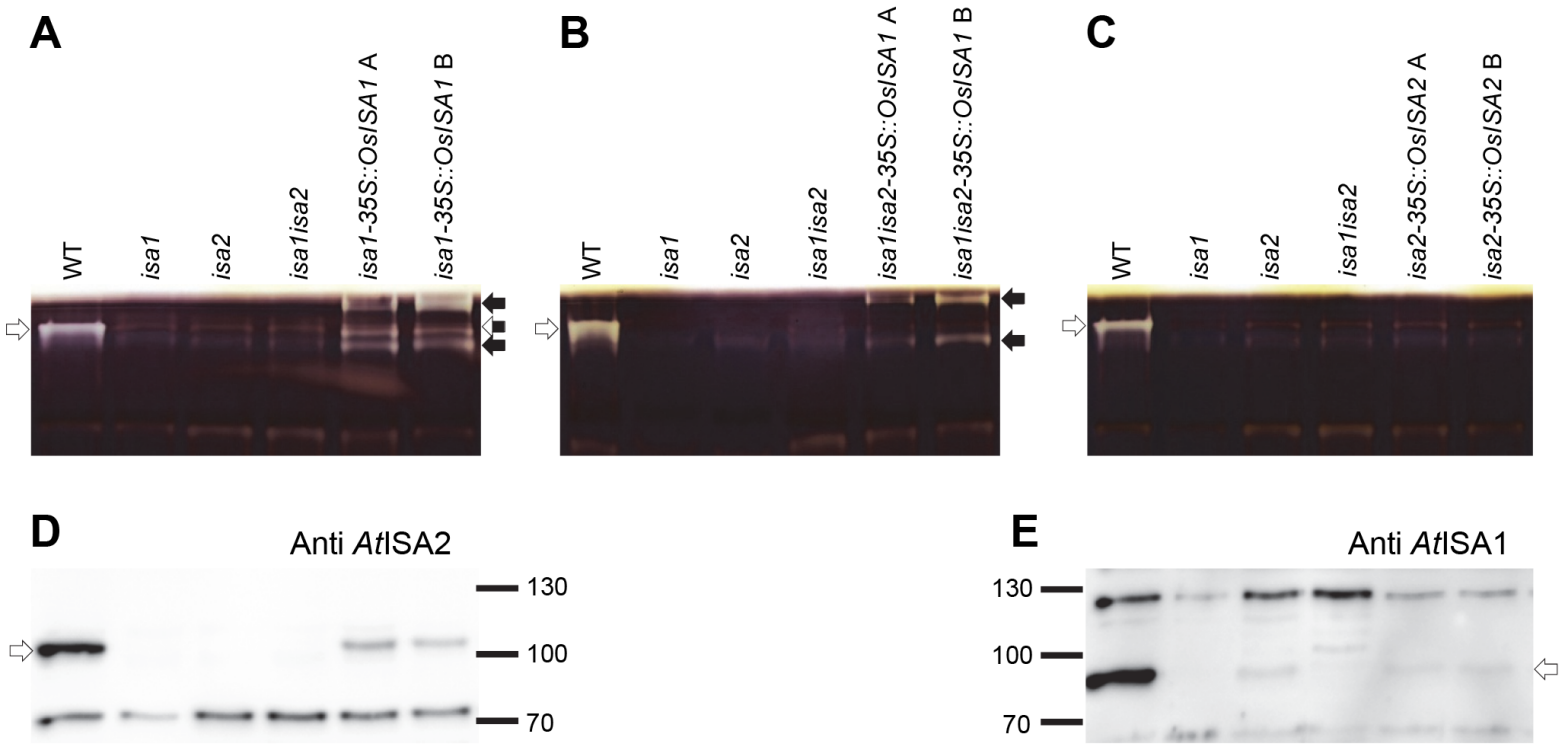
Reconstitution of isoamylase complexes in Arabidopsis isoamylase mutants expressing *OsISA1* and *OsISA2*. A) Soluble protein extracts of the wild type (WT), *isa1*, *isa2*, and *isa1isa2* mutants, and two *isa1* lines complemented with *OsISA1* (*isa1-35S::OsISA1* A and B), analyzed by native PAGE in beta-limit dextrin-containing gels. After electrophoresis and incubation, gels were stained with iodine to reveal bands of activity where the glucan was hydrolyzed. White arrows; the endogenous heteromultimeric Arabidopsis isoamylase (*At*ISA1/*At*ISA2). Black and white arrow; the chimeric Arabidopsis/rice isoamylase (*Os*ISA1/*At*ISA2). Black arrows; the homomutlimeric rice isoamylase (*Os*ISA1). B) Soluble protein extracts of controls (as in A) and two *isa1isa2* lines complemented with *OsISA1* (*isa1isa2-35S::OsISA1* A and B). Arrows as described in A. C) Soluble protein extracts of controls (as in A) and two *isa2* lines complemented with *OsISA2* (*isa2-35S::OsISA2* A and B). Arrow as described in A. D) *At*ISA2 (arrow), detected by immunoblotting (same sample order as in A). Note that *At*ISA2 is not detectable in *isa1*, *isa2*, *isa1isa2* mutants, but is visible in the transformed lines. E) *At*ISA1 (arrow), detected by immunoblotting (same sample order as in C). Note that *At*ISA1 amount is reduced in the *isa2* mutant, but does not increase in the transformed lines.

The complementation of the Arabidopsis mutants suggests that the rice proteins are functional and can substitute the loss of the endogenous proteins. To validate this further we used native PAGE with gels containing beta-limit dextrin, a good substrate to visualize isoamylase enzyme activity. The three isoamylase mutants, *isa1*, *isa2* and *isa1isa2* lacked the debranching activity found in wild-type plants (Fig. 3 and [16]). In *isa1isa2-35S::OsISA1* lines two new activities were visible compared with the *isa1isa2* parental line (Fig. 3B). This is consistent with the idea that two homomultimeric forms of *Os*ISA1 with different electrophoretic mobilities are formed [29]. Interestingly, in *isa1-35S::OsISA1* lines, three new enzyme activities were visible compared with the *isa1* parental line. Two migrated at positions comparable to the activities observed when *OsISA1* was expressed in the *isa1isa2* double mutant (i.e. likely homomultimers of *Os*ISA1) while the third ran at the same height as the endogenous Arabidopsis ISA1/ISA2 complex (Fig. 3A). This is most likely a chimeric isoamylase heteromultimer comprised of *Os*ISA1 and *At*ISA2 subunits. Surprisingly, in the *isa2-35S::OsISA2* lines, no additional enzyme activities were detected compared to the *isa2* parental line. This was unexpected because even though there was phenotypic variability, these plants clearly differed from the *isa2* mutants in respect to starch and phytoglycogen content and more closely resembled the wild type (See Fig. 2).

### Effect of the rice isoamylases on endogenous isoamylase protein levels

In Arabidopsis, only the heteromultimeric ISA1/ISA2 complex occurs and, in mutant plants lacking either ISA1 or ISA2, the activity is abolished (see Fig. 3). Furthermore, evidence suggests that the remaining protein is unstable in the absence of its partner. Thus, ISA1 protein is strongly reduced in amount in *isa2* mutants and ISA2 protein is undetectable in *isa1* mutants [15], [16]. Given this profound effect of one protein on the stability of its interaction partner, we tested what impact the expression of the rice isoamylases has on the levels of the endogenous Arabidopsis proteins.

The same protein extracts used for the native PAGE were analysed by SDS-PAGE and immunoblotting, probing with specific antibodies raised against *At*ISA1 [16] and *At*ISA2 [15]. We confirmed that the ISA1 protein was reduced in *isa2* mutants and the absence of ISA2 protein in *isa1* (Fig. 3 D and E). In *isa1-35S::OsISA1* lines, the Arabidopsis ISA2 protein reappeared, although not to wild-type levels (Fig. 3D). This observation is in agreement with the native PAGE (Fig. 3A), where a putative chimeric *Os*ISA1/*At*ISA2 heteromultimer was detected. It is worth noting that the activity of this putative chimeric enzyme was less intense than that of the endogenous ISA1/ISA2, consistent with the lower levels of *At*ISA2 protein. Nevertheless, these observations strongly suggest that, in these plants, the *Os*ISA1 protein interacts with and stabilises the Arabidopsis ISA2 protein.

In contrast, no increase in the *At*ISA1 protein could be detected in *isa2-35S::OsISA2* compared to the *isa2* parental line (Fig. 3E). It is possible that the residual *At*ISA1 in *isa2* is available to form an active chimeric heteromultimer composed of *At*ISA1 and *Os*ISA2 subunits. This would explain the observed partial complementation of the *isa2* phenotype in these lines (Fig. 2B). However, our inability to detect isoamylase activity in these lines renders this explanation somewhat speculative.

### Starch structure in Arabidopsis isoamylase mutants expressing rice isoamylases

**Figure 4:**
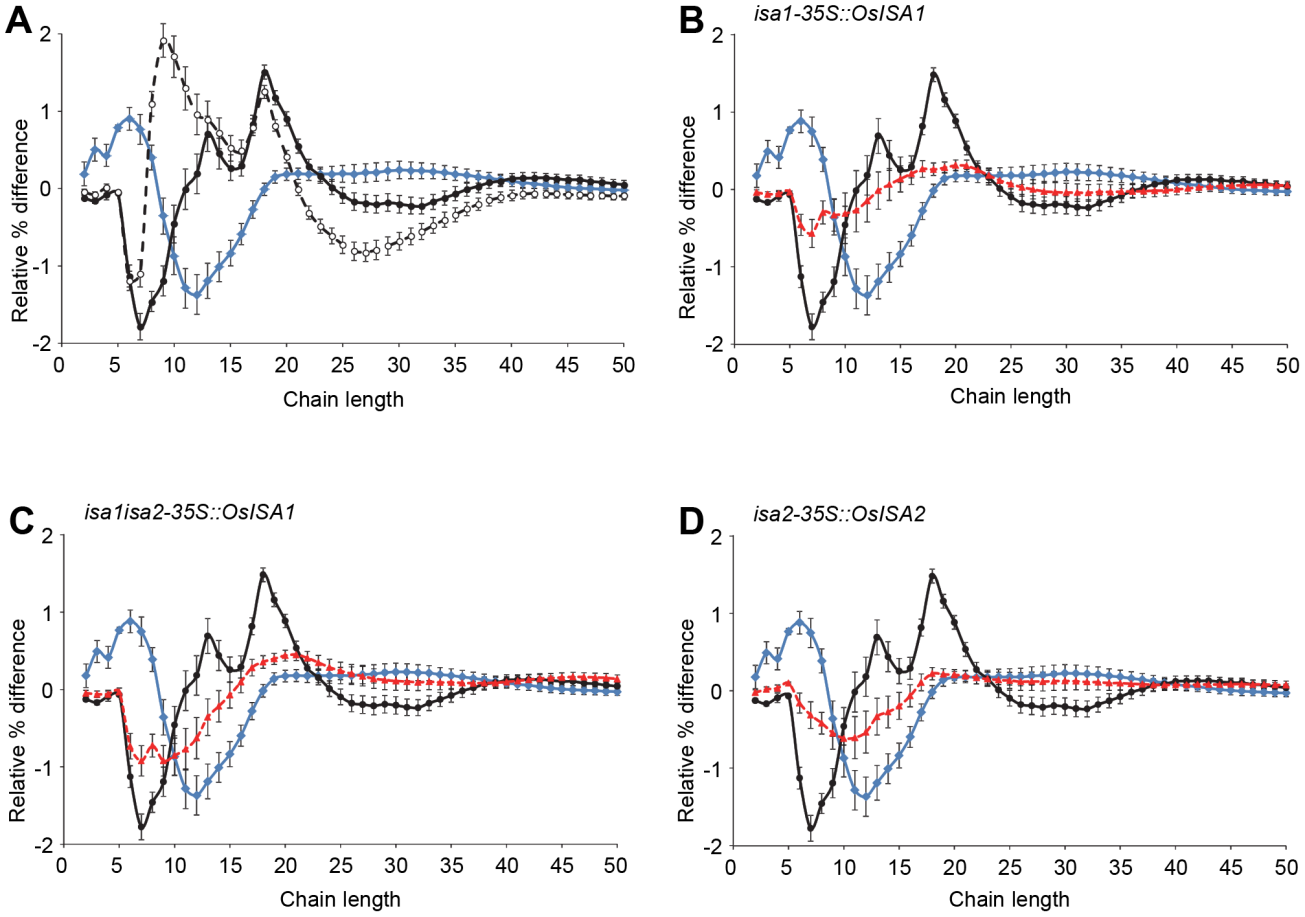
Complementation of the altered amylopectin structure in Arabidopsis isoamylase mutants transformed with rice isoamylases. Starch was extracted from replicate plant samples (as used in Fig. 2B), and the amylopectin CLDs were determined (see Materials and Methods and Fig. 1). The difference plots presented were calculated by subtracting the relative percentage for each chain length of Arabidopsis wild-type amylopectin from the equivalent percentage for the amylopectin from the lines indicated. Errors are calculated from the SE of two compared samples, considering the error law of Gauss. A) Difference plots for amylopectins from *isa1isa2* (blue diamond), rice transient starch (filled black circle) and rice storage starch (open black circle). Note that the CLD of *isa1isa2* amylopectin resembles those of *isa1* and *isa2* single mutants (not shown). Therefore the *isa1isa2* difference plot and that of rice transient starch are used as references in panels B to D. B) Difference plots of *isa1-35S::OsISA1* lines relative to the wild type (red triangle). C) Difference plots of *isa1isa2-35S::OsISA1* lines relative to the wild type (red triangle). D) Difference plots of *isa2-35S::OsISA2* lines relative to the wild type (red triangle). Note that in B to D, the red lines do not simply revert to zero, which would be equivalent to wild-type Arabidopsis amylopectin. They differ more from *isa1isa2* amylopectin (blue diamond) and, to an extent, approach the CLD of rice transient starch (filled black circle).

In addition to the accumulation of phytoglycogen, loss of the ISA1/ISA2 isoamylase in Arabidopsis results in alterations of the CLD of the residual starch (Fig. 4A and [13], [16]). We analysed starch from plants harvested at the end of the day (the same samples as used for the starch measurements; Figure 2B). CLD profiles were calculated by averaging the three biological replicates of each genotype. As previously reported, the amylopectin CLDs from the starch from all three Arabidopsis *isa* mutants (*isa1*, *isa2* and *isa1isa2*) were the same, having more very short chains (d.p. 4 to 7) but less medium-length chains (d.p. 10 to 16) compared to the wild type (only *isa1isa2* is shown). We compared the amylopectin CLDs of the starch synthesized in our Arabidopsis mutants complemented with the rice isoamylases to the wild type, to the *isa1isa2* double mutant, and to the rice starches. For each transformation, the three independent lines had very similar CLD profiles (Fig. S1). Both the *isa1-35S::OsISA1* and the *isa2-35S::OsISA2* lines synthesized starch more similar to wild-type Arabidopsis starch than to *isa1isa2* mutant (Fig. 4 B and D). Interestingly, the *isa1isa2-35S::OsISA1* CLD profile was very different from the *isa1isa2* mutant, and was intermediate between wild type Arabidopsis starch and rice leaf starch (Fig. 4C).

## Discussion

### Interchangeability of rice and Arabidopsis isoamylase subunits

In this work, we demonstrated the interchangeability of enzymes in starch metabolism between monocotyledonous and dicotyledonous plants. Arabidopsis mutants lacking the ISA1/ISA2 complex have reduced capacity to synthesise crystalline starch - instead they accumulate water-soluble phytoglycogen. Here, we show that the rice isoamylase proteins are able to rescue this phenotype. All the transgenic lines analysed contained exclusively starch (Fig. 2B), even though the responsible isoamylase complex differed in each case. In *isa1isa2* expressing *OsISA1*, we observed two complexes (Fig. 3B). In these plants *Os*ISA1 is the sole ISA protein present in these plants, suggesting that these two are homomultimeric *Os*ISA1 complexes. The formation of an *Os*ISA1 complex was expected, as a homo-pentameric form in rice endosperm has been reported [20]. The observation of two complexes in our plants suggests that the *Os*ISA1 protein can also adopt a different oligomeric state, or is post-translationally modified in a way that affects its migration in our gels (i.e. by changing surface charge or substrate affinity). Consistent with our observations, early studies in which the homomultimer was purified to homogeneity from rice endosperm also yielded an enzyme which migrated in several positions on a native gel and in two positions during isoelectric focusing [29]. Importantly, these data show that starch synthesis in Arabidopsis is not dependent on the presence of ISA2, even though this subunit is critical for the stability of the endogenous enzyme.

In the *isa1* lines expressing *OsISA1*, we observed an additional enzymatic activity besides the two homomultimeric *Os*ISA1 complexes (Fig. 3A). The difference compared to *isa1isa2* expressing *OsISA1* is the intact *AtISA2* gene, so the additional activity is most probably a chimeric heteromultimeric isoamylase complex composed of *Os*ISA1 and *At*ISA2 subunits. This is in agreement with the increased *At*ISA2 protein content in these lines, compared to the *isa1* mutants, in which *At*ISA2 protein is not detectable (Fig. 3D; [15]). The formation of a complex between *Os*ISA1 and *At*ISA2 presumably stabilizes the *At*ISA2 protein. This result implies that the ISA1 and ISA2 proteins are sufficiently conserved between the dicot Arabidopsis and the monocot rice to allow assembly into an enzymatically functional complex. This is consistent with the ability of the maize ISA1 to form an active chimeric complex with *At*ISA2 *in vitro* [28].

In the *isa2* lines expressing *OsISA2*, the *isa2* phenotype was rescued and the plants produced exclusively starch and no phytoglycogen. The most likely explanation for this would be that *At*ISA1 and *Os*ISA2 assemble into an active enzyme in these plants, as seen for *Os*ISA1 and *At*ISA2. However, unexpectedly, we were not able to detect an additional isoamylase activity compared to *isa2* mutants (Fig. 3C). It is possible that the activity may be below the detection limit of our native gel assay. Although we tried increasing the protein content five-fold and prolonging the incubation time from 2 h to 12 h, no activity band was observed even under these conditions. We also did not observe a stabilising effect of *OsISA2* expression on *At*ISA1 protein levels (in contrast to the stabilisation of *At*ISA2 by overexpressing *OsISA1*). However, there is residual *At*ISA1 protein in the *isa2* mutants, which was comparable in lines overexpressing *OsISA2*. This might mean that there is sufficient protein to partner *Os*ISA2. It is also possible that a chimeric complex is formed which has an altered migration such that it co-migrates with another starch degrading activity and is therefore masked. Alternatively, the chimeric enzyme may not resolve well under our experimental conditions or may be very labile during extraction.

The idea that a chimeric *At*ISA1/*Os*ISA2 isoamylase complex may be present at a very low abundance in our *isa2-35S::OsISA2* plants would suggest that extremely low levels of a functional isoamylase complex are sufficient to facilitate starch synthesis. Although all three lines had a similar starch structure it is worth noting that the starch level was not the same as in the wild type (Fig. 2B). The fact that very low levels of isoamylase may be functional was also suggested from a study of potato plants in which either *StISA1* or *StISA2* were silenced. Transgenic lines were identified which showed no detectable isoamylase activity on native PAGE (as used here) but still accumulated almost exclusively starch and only tiny amounts of phytoglycogen [17]. This study differs from most, as intermediate levels of the wild-type isoamylase activity were obtained. In contrast, when mutants are used, they generally result in a complete loss of isoamylase activity or an altered isoamylase enzyme, which is typically accompanied by a phytoglycogen-accumulating phenotype (e.g. [6], [16], [18], [21], [22]. These results lead us to speculate that as isoamylase activity is reduced and becomes limiting, the initial phenotype may be a drop in starch production before further reduction in the enzyme results in phytoglycogen accumulation. However, this is an aspect of the phenotype that we cannot fully explain at present.

The fact that the rice proteins were able to functionally replace the Arabidopsis proteins does not necessarily mean that the converse is true, and that the Arabidopsis proteins would be able to complement the phenotypes of the corresponding rice mutants. This may be especially the case for ISA1, because Arabidopsis ISA1 seems unable to form an active homomultimer. This may be important as it is specifically the ISA1 homomultimer that is thought to facilitate starch synthesis in the rice endosperm [25]. Mutants lacking ISA2 have only the homomultimer and produce near-normal starch. In contrast, plants overexpressing ISA2 possess the heteromultimer, but lack the ISA1 homomultimer. These plants produce only half as much starch as the wild type and the starch is aberrant in structure, suggesting that the specificity of the heteromultimer is not ideally matched to the glucan produced by the other starch biosynthetic enzymes of the endosperm [25].

### A role for isoamylase in determining species-specific starch structure?

The replacement of Arabidopsis ISA1 and ISA2 with the rice orthologs enabled us to study the effect of distinct isoamylase complexes on the fine structure of starch. Given that the isoamylases of different subunit compositions have been shown to have distinct functionalities in cereal endosperm, it is reasonable to hypothesise that the differences in amylopectin between species may be partly dependent on the isoamylases they contain. All three transgenic lines had starch structures that were similar, but not identical, to Arabidopsis wild-type starch (Fig. 4). The line possessing only the rice homomultimers had the most divergent amylopectin structure. The increased abundance of short chains between d.p. 3 and d.p. 8 in the *isa1isa2* mutant CLD (compared to wild-type Arabidopsis amylopectin) was lost. Instead an increased abundance of longer chains between d.p. 6 and d.p. 14 was seen. This structure is somewhat intermediate between Arabidopsis and rice transient starch. In the *isa1-35S::OsISA1* lines the amylopectin CLD was much more similar to wild-type Arabidopsis amylopectin, but the same tendencies towards increased number of chains between d.p. 6 and d.p. 14 was seen. In this line, the homomultimeric *Os*ISA1 is accompanied by a chimeric Arabidopsis/rice isoamylase complex - *Os*ISA1/*At*ISA2. This chimeric enzyme presumably functions in a similar way to the endogenous Arabidopsis enzyme. Plants putatively containing the other chimeric Arabidopsis/rice isoamylase complex - *At*ISA1/*Os*ISA2 also had starch with modest increases in the numbers of chains between d.p. 6 and d.p. 14. It has been proposed that the catalytically inactive subunit ISA2 has a regulatory function, provides substrate specificity, or increased stability to the catalytic ISA1 subunit [5]. These results suggest that the replacement of the *At*ISA2 with *Os*ISA2 causes a change in substrate specificity, but this conclusion needs to be treated with caution since no activity was directly measured. Further analysis, such as X-ray scattering, scanning electron microscopy and differential scanning calorimetry could be used to study in more depth the structure and properties of starch produced in plants with different chimeric isoamylase complexes.

## Conclusion

Our results are consistent with the findings of other studies. The wheat ISA1 (*Ta*ISA1) protein was able to revert the phytoglycogen-accumulating phenotype of the rice *sugary-1* mutant (lacking *Os*ISA1) back to a starch-accumulating phenotype [30]. However, rice and wheat are much more closely related to each other than rice and Arabidopsis used, and wheat may also have the two types of isoamylase complex (i.e. homo- and heteromultimers). Nevertheless, after complementation with *Ta*ISA1, the amylopectin CLD was similar – but not identical – to the wild type rice amylopectin. Recently, it was shown that the maize ISA1 (*Zm*ISA1) protein expressed in the Arabidopsis *isa1* and *isa1isa2* mutants were able to restore exclusive starch synthesis in Arabidopsis, similar to our observations. Interestingly, the authors observed a greater variability of the starch content, with some transgenic lines producing even more starch than the wild type, which was not the case in our plants. However, this raises the possibility that the isoamylase may somehow determines not only the starch structure but also the amount produced. Interestingly, the ZmISA1 expressing plants produced starch with the same amylopectin CLD as the Arabidopsis wild type [28]. This is in contrast to our results, but might be explained by the fact that the amylopectin CLDs of maize and Arabidopsis are more similar to each other than Arabidopsis and rice (Fig. 1). It is tempting to conclude from all of these studies that isoamylase can contribute to species-specific amylopectin structure. However, it is important to note that the differences in structure that we measure are relatively small. Therefore, it seems likely that species-specific differences in the starch synthases and branching enzymes might also contribute.

Furthermore, it is important to emphasise that differences in expression of these enzymes between tissues or between species may have an equally large impact on the final structure of amylopectin. This was strikingly illustrated recently through RNA-seq analysis of the myrmecophytic tropical tree *Cecropia peltata*, where differential expression of the starch-biosynthetic apparatus results in amylopectin production in leaves and glycogen biosynthesis in food bodies produced to feed mutualistic ants [31].

## Materials and Methods

### Plants and growth conditions

*Arabidopsis thaliana* plants were grown in Percival AR95 climate chambers at constant 20°C, 60% relative humidity, 12-h photoperiod and a light intensity of 150 μmol photons m^−2^ sec^−1^. Seeds were sown out on soil and seedlings transferred to individual pots two weeks after germination. Mature plants were harvested three weeks later, weighed, frozen in liquid N_2_ and kept at –80°C until use. Single *isa* mutants used were described in earlier studies: *isa1*, *isa1-1*, SALK_042704 [16]; *isa2*, *isa2-1* (initially called *dbe1-1* [32] and the double mutant *isa1isa2*, *isa1-1/isa2-1* [16].

Rice (*Oryza sativa* cv. Taipei 309) plants were grown in greenhouse at 23°C, 70% relative humidity at night and 26°C, 80% relative humidity at day with a minimum 14-h photoperiod and minimum light intensity of 250 μmol photons m^−2^ sec^−1^. Leaves for the extraction of transient starch were harvested from four-week-old plants. Mature, dry seeds from these plants were used for storage starch purification. Potato (*Solanum tuberosum* cv. Agata) tubers were obtained from a local market and used for storage starch purification. For transient starch isolation, tubers were sprouted and leaves harvested after four weeks of growth in a chamber (Kälte 3000 AG, Landquart, Switzerland) at constant 22°C, 70% relative humidity, with a 12-h photoperiod and a light intensity of around 250 μmol photons m^−2^ sec^−1^. Maize (*Zea mays* Benicia, Pioneer) plants were grown in a chamber (Kälte 3000 AG) at constant 22°C, 70% relative humidity with a 12-h photoperiod and a light intensity of around 250 μmol photons m^−2^ sec^−1^. Leaves for transient starch were harvested from four-weeks-old plants. Purchased seeds were used directly for storage starch purification.

### SDS-PAGE blotting and native PAGE

Soluble proteins were extracted from leaves (150 mg) of five-week-old plants by homogenization in 1 mL of medium containing 100 mM MOPS, pH 7.2, 1 mM EDTA, 5 mM DTT, 10% (v/v) glycerol, 1 x Complete Protease Inhibitor Cocktail (Roche), using all-glass homogenizers. Homogenates were subject to centrifugation (16,000 *g*, 10 min, 4°C). Proteins were separated by SDS-PAGE using standard methods, transferred to PVDF membranes, probed with antibodies against *At*ISA1 [16] and *At*ISA2 [15], and developed using Immun-Star horseradish peroxidase chemiluminescence (Bio-Rad). For native PAGE, proteins were separated with gels containing 6% (w/v) polyacrylamide and 0.3% (w/v) β-limit dextrin (Megazyme, Bray, Ireland). After electrophoresis, gels were incubated for 2 h at 37°C in medium containing 100 mM Tris-HCl, pH 7.2, 1 mM MgCl_2_, 1 mM CaCl_2_ and 5 mM DTT. Reaction were stopped and activities visualized by application of Lugol solution (5% [w/v] I_2_, 10% [w/v] KI).

### Extraction and measurement of glucose polymers

The extraction procedure was the same for all plant species and tissues and was performed using perchloric acid as described previously [16]. Glucose polymers (starch and phytoglycogen) were quantified by enzymatic digestion with amyloglucosidase (*Aspergillus niger*; Roche) and α-amylase (*pig pancreas*; Roche), and released glucose was measured spectrophotometrically [33].

### Structural analysis of starch and phytoglycogen

Starch and phytoglycogen in the insoluble and soluble fractions from the perchloric acid-extracted plant material, respectively, were used for glucan structural analysis. Volumes containing 100 μg of starch or phytoglycogen were used from each of four individually extracted plants. Samples were boiled for 10 min prior enzymatic digestion. Debranching reactions with *Pseudomonas amyloderamosa* isoamylase (Sigma-Aldrich, Buchs, Switzerland) and *Klebsiella planticola* pullulanase (Megazyme) were carried out for 2 h at 37°C in 10 mM Na-acetate, pH 4.8. The resulting linear glucans were analysed by HPAEC-PAD as described previously [8].

### Construction of *OsISA1* and *OsISA2* overexpression vectors and plant transformation

*Os*ISA1 and *Os*ISA2 coding sequences were amplified by PCR using attB-site containing primers (Table S1). The templates were pGEM-T Easy vectors containing *OsISA1* and *OsISA2* cDNA sequences [25]. The PCR fragments were cloned into pDONR 221 via the BP reaction of the Gateway recombination cloning technology (Invitrogen, LuBioScience GmbH. Lucerne, Switzerland). After sequence conformation, inserts were cloned into the plant vector pB7WG2 [27] using the Gateway LR reaction. Stable *Arabidopsis thaliana* transgenic lines were generated through *Agrobacterium tumefaciens*-mediated transformation using the floral dip method [34]. Independent transformants were selected on soil by spraying two-week-old plants with 0.1% (v/v) BASTA herbicide.

## Supporting Information

Figure S1 **Amylopectin structure of starch produced in independent** ***isa1-35S::OsISA1*** **,** ***isa2-35S::OsISA2*** **and** ***isa1isa2-35S::OsISA1*** **lines.** Comparison of the three independent transformants lines of *isa1-35S::OsISA1*, *isa1*isa2*-35S::OsISA1* and *isa2-35S::OsISA2* illustrating the uniformity in starch structure produced. Raw data used to calculate the mean normalized CLD (± SE, n = 3) was also used to calculate the difference plots in Fig. 5 B to D. A) CLDs of the lines *isa1-35S::OsISA1* A (open circle), *isa1-35S::OsISA1* B (open square) and *isa1-35S::OsISA1* C (open triangle). B) CLDs of the lines *isa1isa2-35S::OsISA1* A (open circle), *isa1isa2-35S::OsISA1* B (open square) and *isa1isa2-35S::OsISA1* C (open triangle). C) CLDs of the lines *isa2-35S::OsISA2* A (open circle), *isa2-35S::OsISA2* B (open square) and *isa2-35S::OsISA2* C (open triangle).
(TIF)

Table S1 **Primers used for Gateway cloning of** ***OsISA1*** **and** ***OsISA2.***
(DOCX)
