# Supplementary figures and images for: Replacement of the Endogenous Starch Debranching Enzymes ISA1 and ISA2 of Arabidopsis with the Rice Orthologs Reveals a Degree of Functional Conservation during Starch Synthesis
Source: PLoS One. 2014 Mar 18;9(3):e92174. doi: 10.1371/journal.pone.0092174 (PMC3958451; doi:10.1371/journal.pone.0092174)

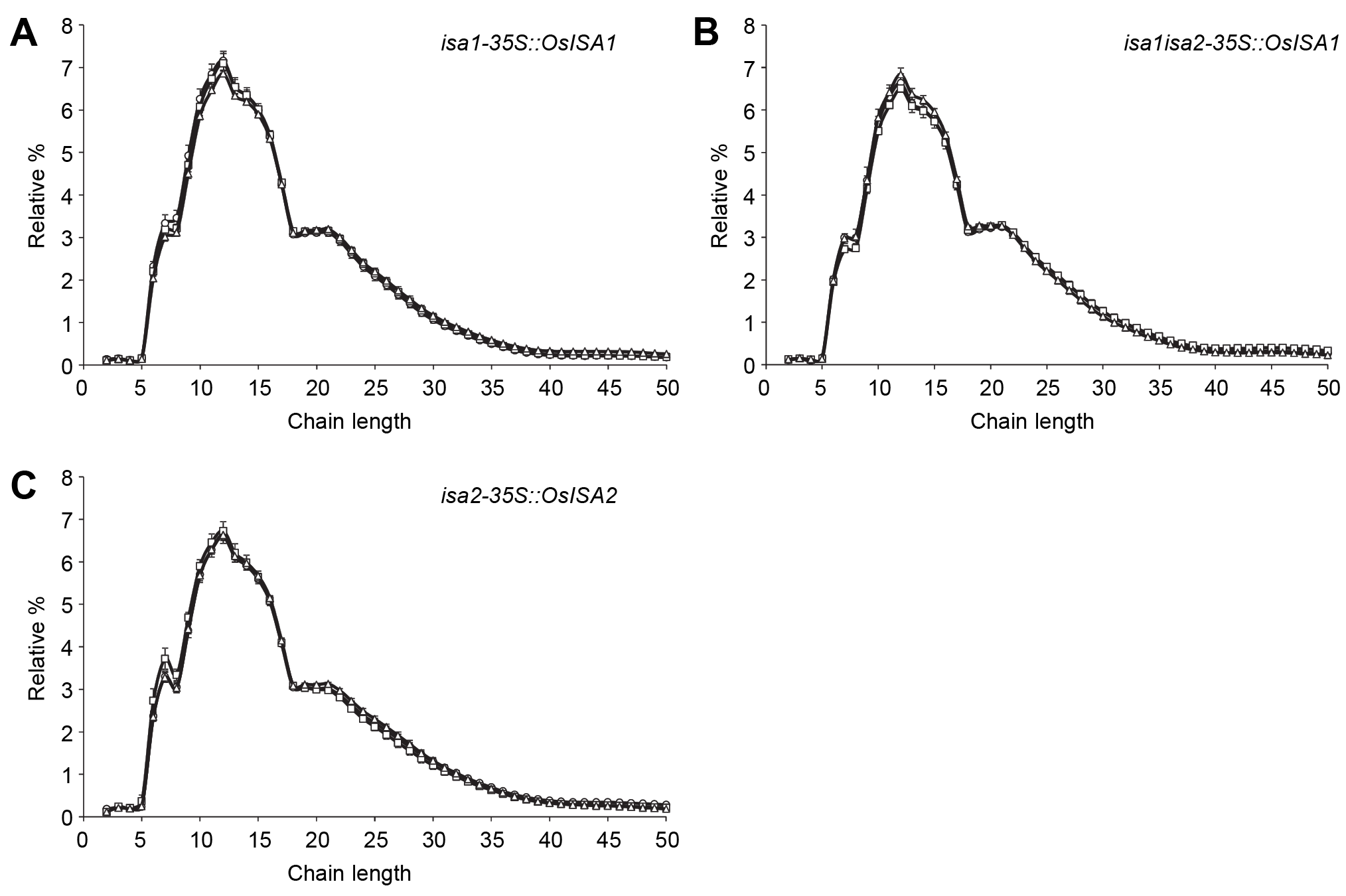

Supplement: Figure S1 — Amylopectin structure of starch produced in independent isa1-35S::OsISA1 , isa2-35S::OsISA2 and isa1isa2-35S::OsISA1 lines. Comparison of the three independent transformants lines of isa1-35S::OsISA1, isa1isa2-35S::OsISA1 and isa2-35S::OsISA2 illustrating the uniformity in starch structure produced. Raw data used to calculate the mean normalized CLD (± SE, n = 3) was also used to calculate the difference plots in Fig. 5 B to D. A) CLDs of the lines isa1-35S::OsISA1 A (open circle), isa1-35S::OsISA1 B (open square) and isa1-35S::OsISA1 C (open triangle). B) CLDs of the lines isa1isa2-35S::OsISA1 A (open circle), isa1isa2-35S::OsISA1 B (open square) and isa1isa2-35S::OsISA1 C (open triangle). C) CLDs of the lines isa2-35S::OsISA2 A (open circle), isa2-35S::OsISA2 B (open square) and isa2-35S::OsISA2 C (open triangle). (TIF) [file pone.0092174.s001.tif]
