# Supplementary material for: Replacement of the Endogenous Starch Debranching Enzymes ISA1 and ISA2 of Arabidopsis with the Rice Orthologs Reveals a Degree of Functional Conservation during Starch Synthesis
Source: PLoS One. 2014 Mar 18;9(3):e92174. doi: 10.1371/journal.pone.0092174 (PMC3958451; doi:10.1371/journal.pone.0092174)
Supplement: Table S1 — Primers used for Gateway cloning of OsISA1 and OsISA2. (DOCX) [file pone.0092174.s002.docx]

**Table S1**

**Table S1. Primers used for gateway cloning of *OsISA1* and *OsISA2*.**

| Name | Sequence (5`-3`) |
| --- | --- |
| AttB1-OsISA1 | GGGGACAAGTTTGTACAAAAAAGCAGGCTTCACCATGGCGAGCCTCCCGCACTG |
| AttB2-OsISA1 | GGGGACCACTTTGTACAAGAAAGCTGGGTCATCATCAGGCTGCAATTCAAGGATG |
| AttB1-OsISA2 | GGGGACAAGTTTGTACAAAAAAGCAGGCTTCACCATGGCGTCCCTCCCCG |
| AttB2-OsISA2 | GGGGACCACTTTGTACAAGAAAGCTGGGTCGGCAAGAGCGCTCTTTGATTC |
